# Supplementary material for: MetaRibo-Seq measures translation in microbiomes
Source: Nat Commun. 2020 Jun 29;11:3268. doi: 10.1038/s41467-020-17081-z (PMC7324362; doi:10.1038/s41467-020-17081-z)
Supplement: Supplementary file 10 — Supplementary Data 7 [file 41467_2020_17081_MOESM10_ESM.zip › File2/Confidence_High_Taxonomy/155553_out.krona.html]

Javascript must be enabled to view this page.

members
magnitude
magnitudeUnassigned
count
unassigned
taxon
rank

155553\_out

8

2
8
superkingdom

1239
5
phylum

5
1263017
species

SRS012969\_contig\_number\_39458SRS023526\_contig\_number\_10466SRS055017\_contig\_number\_28777SRS077392\_contig\_number\_42331SRS077849\_contig\_number\_14085

3
544448
phylum

class
31969
3

3
186329
order

2053494
3

SRS011134\_contig\_number\_11803SRS022609\_contig\_number\_20383SRS098571\_contig\_number\_46745
species
